# Does glochidia infestation of the freshwater pearl mussel (*Margaritifera margaritifera*) affect foraging behavior and growth in brown trout (*Salmo trutta*)?

**DOI:** 10.1007/s00436-026-08759-2

**Published:** 2026-09-16

**Authors:** Niklas Wengström, Johan Höjesjö, Karl Filipsson, Lisa Loeb, Hampus Kvarnliden, Martin Österling

**Affiliations:** 1 Department of Biological and Environmental Sciences, University of Gothenburg, Gothenburg, SE 405 30 Sweden; 2 Swedish Anglers Association, Sjölyckan 6, Gothenburg, SE 416 55 Sweden; 3 Institution for Environmental and Life Sciences, Karlstad University, Karlstad, SE 651 88 Sweden

**Keywords:** Behaviour, Unionid mussels, Prey handling, Host, Parasite

## Abstract

Fish serve as hosts to a wide range of parasites, which can affect them in many ways, including their survival, growth, and foraging behavior. The highly threatened unionid mussels have an ectoparasitic life stage on the gills, skin, or fins of fish. Here, we compared prey handling time and growth between groups of brown trout (*Salmo trutta*) that were infested and non-infested with glochidia larvae of the freshwater pearl mussel (*Margaritifera margaritifera*). In a prey handling test, individual trout were presented with one large prey (maggot) and one small prey (*Gammarus pulex*) daily over eight days. Compared to non-infested trout, prey handling time was longer for infested trout feeding on larger but not on smaller prey. In a growth test, where trout were fed daily with minced calf liver over nine weeks, the growth rate was lower for infested than for non-infested trout. Moreover, the growth rate was higher for infested trout from which glochidia were expelled compared to trout from which glochidia were not expelled. We suggest that glochidia infestation can increase prey handling time of fish, ultimately reducing fish growth, but that slow prey handling may be transitory for fish losing glochidia, and result in compensatory growth of the fish. Slow prey handling, in combination with a negative effect of glochidia infestation on food competition between infested and non-infested trout, may be reasons behind the lower growth of infested fish.

## Introduction

Parasite-host interactions have shaped some of the most exciting biotic interactions on the planet, and the evolutionary arms race between the species involved is an important factor driving adaptation and speciation (Poulin 2007; Stern and Sorek 2010). The coevolution between parasites and hosts has resulted in a variety of life cycles and life history strategies that enhance parasite transmission within hosts, and host defence mechanisms to avoid parasitic infections (Poulin 1998; Moore 2002). By definition, parasites impose costs that reduce host fitness; in mammals, these costs often manifest as decreased fecundity and reduced survival rates, as demonstrated in red deer *Cervus elaphus* (Albery et al. 2021). Examples from fish include parasitic nematodes that decrease the fecundity of the Western mosquitofish (*Gambusia affinis*) (Deaton 2011) and the cestode *Schistocephalus solidus*, which reduces reproductive investment in female three-spined sticklebacks (*Gasterosteus aculeatus*) (Schultz et al. 2006).

Fish serve as hosts to a variety of parasites with different life cycle strategies, from complex life cycles (indirect) with more than one host to complete the life cycle to less complex life cycles (direct) with one host (Poulin 2007). Both life cycle strategies can negatively affect the growth, survival, and reproduction of infested fish (Barber et al. 2000). Several studies have reported fitness-related costs, such as reduced growth rates, e.g., rainbow smelt (*Osmerus mordax*) (Sirois and Dodson 2000) infected with *Proteocephalus sp*, three-spined sticklebacks infected with *Schistocephalus solidus* (Pennycuick 1971), and sockeye salmon (*Oncorhynchus nerka*) infected with *Eubothrium salvelini* (Boyce 1979). On the other hand, the parasite intensity and growth rate of the Arctic charr (*Salvelinus alpinus*) were positively related, but the parasitic load on the Arctic charr was probably caused by their increased consumption rate of parasitized *Gammarus lacustris* (Henriksen et al. 2019).

The highly threatened unionid mussels are temporary ectoparasites on one or more fish species (Graf and Cummings 2007). The unionid mussels have a direct parasitic life cycle that includes four stages: the egg stage, the larval (glochidium) stage, the juvenile mussel stage, and the adult mussel stage. The parasitic glochidia larvae attach to the fins, body, and/or gills of a host fish, and are encysted by the fish (Arey 1931; Blažek and Gelnar 2006). Different mussel species parasitize their host fish for weeks to over a year, and during this time, they can negatively affect the fish (Horký et al. 2014). For example, the infestation intensity of the kidneyshell mussel (*Ptychobranchus occidentalis*) was suggested to negatively affect the body volume of the rainbow darter (*Etheostoma caeruleum*) (Crane et al. 2011).

The freshwater pearl mussel (*Margaritifera margaritifera*) parasitizes brown trout (*S. trutta*) and/or Atlantic salmon (*Salmo salar*) for 10–12 months (Bauer 1987; Karlsson et al. 2014). Several studies have suggested that glochidia infestations can have negative effects on trout physiology and behavior. Spleen enlargement and longer time to reach basal ventilation rates, possibly because of thicker gills, have been observed in artificially infested trout (Thomas et al. 2013). The swimming speed of trout was lower in glochidia-infested trout compared to a non-infested control group (Taeubert and Geist 2013). Reduced drift-feeding rate, activity, and aggression, and increased metabolic rate and haematocrit levels were found for naturally infested compared to non-infested trout (Österling et al. 2014; Filipsson et al. 2016, 2017). In a controlled growth experiment over 45 weeks, Chowdhury et al. (2019) found that infested trout had a lower growth rate compared to a control group of non-infested trout. Lastly, the effect of glochidia infection of *Margaritifera laevis* has been shown to continue after the glochidia have fallen off the host *Oncorhynchus masou masou* (Ooue et al. 2017). Such effects may also lead to ecological changes, such as altered species interactions, population growth, or changes in migration behaviour in streams and rivers (Rock et al. 2025).

In the present study, we aimed to extend existing knowledge by investigating whether glochidia infestation affected prey handling and whether the loss of glochidia affected host fish growth. We examined differences in prey handling time and growth between infested and non-infested juvenile brown trout. We believe the thicker gills of infested trout physically interfere with feeding, and we therefore hypothesized that prey handling time for trout held individually would be longer for infested than for non-infested trout. We also hypothesized that growth would be lower for infested than for non-infested trout in groups including both infested and non-infested trout. Lastly, we hypothesized that growth would be higher in infested trout that lost glochidia over time compared to infested trout where glochidia remained on the fish during the experimental period.

## Methods

### Fish collection and husbandry

We collected 1 + brown trout (*Salmo trutta*) from river Slereboån (Lat: 58.00928, Long: 12.342882) in the Göta Älv catchment on the 14^th of^ September 2015 using electrofishing (LR-20B, Smith-Root, Vancouver, WA, USA). The population of *M. margaritifera* is estimated to contain > 5000 individuals (Martinsson and Wengström 2014; Wengström et al. 2022), and the abundance of mussels in the area where the trout was caught was 0.47 mussels/m^2^. All trout were anesthetized (MS-222, 50 mg/L, Pharmac Ltd, United Kingdom) in the field and checked for glochidia encystment using a stereo magnifier (Wild, Heerbrugg, Switzerland). A total of 38 infested and 99 non-infested trout were transported in well-aerated tanks to the zoology building at the Department of Biological and Environmental Sciences, University of Gothenburg. The infested and non-infested trout were kept separately in two 120-liter aquaria (0.64 × 0.48 × 0.40 m) with flow-through filtration systems and substrates consisting of gravel (2–10 mm). The photoperiod followed natural daylight cycles, and the water temperature was kept at 12 °C. Shelter for the trout was created by adding dark opaque plastic boards. Before the experiment, the trout were fed with maggots, *Calliphoria vomitaria* (~ 1% of the trout’s body mass) twice a week.

### Prey handling experiment

After 11 days of acclimatization, we randomly chose 14 infested (116 mm ± 4.6 SE, 17.9 g ± 1.92SE) and 14 non-infested trout (117 mm ± 3.3SE, 17.08 g ± 1.29SE) to assess the effect of glochidia infection on trout prey handling time. Neither trout length nor weight differed statistically between infested and non-infested fish (t-test, *p* = 0.92 and *p* = 0.76, respectively). We relocated all trout to individual experimental 40-L aquarium compartments (0.35 × 0.35 × 0.35 m). The experimental aquaria were connected to the same flow-through water system as the holding aquaria, and the gravel (2–10 mm) was cleaned of detritus and potential food items before the trout were gently placed in the aquaria. After 24 h in the aquaria, each trout was simultaneously presented with one live maggot and one live *Gammarus pulex* to habituate the fish to the different prey used in the experiment. The size of the maggots and *G. pulex* were between 15 and 20 mm in length. We thereafter let the trout acclimatize for 3 days before starting the trials.

As the experiment commenced, each trout was presented with one live maggot and one live *G. pulex* every day for eight consecutive days. Using pincers, we gently placed the prey on the water surface in the middle of the aquarium. One prey was given in the morning and the other in the afternoon. The order in which the prey was given each day was randomized between days, as was the order in which we tested each trout. We video recorded all foraging trials (Sony HDR-XR155) to simplify and verify data collection. When all trials were done, we carefully netted the trout, anesthetized them (2-phenoxyethanol, 0.5 ml/l, Aldrich chemistry, Germany), and measured fork length and mass of all trout. We also controlled glochidia encystment on all fish during the anaesthesia by gently lifting the operculum on both sides of the trout and counting the glochidia using a stereo magnifier (Wild, Heerbrugg, Switzerland). All infested trout had a glochidia infection of > 50 glochidia, while the non-infested trout lacked glochidia infestation completely. All trout were released in river Slereboån when the experiment was finalized.

### Growth experiment

After seven days of acclimatization, we randomly chose 24 infested (104 ± 1.8, 86–117 mm, 12.1 ± 0.58, 6.6–17.3 g) and 24 non-infested (105 ± 1.5, 93–118 mm, 12.3 ± 0.54, 8.6–16.7 g) trout from the holding aquaria for the growth experiment. Trout size did not differ between infested and non-infested trout (t-test, *p* = 0.66 and *p* = 0.76 for fork length and wet mass, respectively). All trout were measured and marked with 12 mm passive integrated transponder (PIT) tags (HDX ISO 11784/11785, OREGON RFID, Portland, OR, U.S.A.) at the lab. All trout were treated carefully while anesthetized (2-phenoxyethanol, 0.5 ml/l, Aldrich Chemistry, Germany) whereupon PIT-tags were inserted into the coelomic cavity of each trout by the use of a syringe. Trout were thereafter divided into eight groups. Each group consisted of three infested and three non-infested size-matched trout (variation in mass ≤ 3.6 g and in length ≤ 13 mm). Each group of trout was placed in a 100-L aquarium (0.65 × 0.5 × 0.3 m). The substrate in each aquarium consisted of sand, gravel, and rocks. To mimic a natural stream structure, black plastic boards, plastic tubes, and plastic plants were added to each aquarium. Half of the area of each aquarium was covered with transparent glass, while the other half was covered with both glass and a black screen, and all four sides of each aquarium were covered with black screens. The aquaria were supplied with freshwater from a flow-through filtration system. Photoperiod followed natural daylight cycles, and the water temperature was kept constant throughout the experiment at 12 °C.

Trout were fed 1.25% of their body mass on two occasions every weekday (Monday - Friday) for 9 weeks, using minced calf liver as food. After 3, 6, and 9 weeks (on Mondays, after being without food for two days), all trout individuals were anesthetized (2-phenoxyethanol, 0.5 ml/l, Aldrich Chemistry, Germany), whereupon they were measured and weighed (fork length to mm and body mass to 0.01 g), and the glochidia were counted. When these measurements had been done, all the trout were placed back into their original aquarium. In accordance with the changes in body mass over time, we adjusted the amount of feed given after each measuring occasion. Six trout, four infested and two non-infested from three different aquaria, died before the experiment was finalized and were excluded from all analyses. After nine weeks, when the experiment ended, we released all the trout back into river Slereboån.

## Data collection and statistical analyses

### Prey-handling experiment

From the video footage, we calculated the arithmetic mean of prey-handling time in seconds for both maggots and *G. pulex*, for each trout that had foraged on each prey species at least two times. Time was measured between when the trout took the prey and when the prey was consumed. In total, this included 11 infested and 12 non-infested trout for maggots and 11 infested and 7 non-infested trout for *G. pulex*. We analysed the prey-handling data with generalized linear models, using a gamma distribution with a log-link function. We generated one model for the prey-handling time of maggots and one for *G. pulex*. We included prey-handling time in seconds as a dependent variable, infested/ non-infested trout as a fixed factor, and trout wet mass (g) as a covariate in the models.

### Growth experiment

We calculated the relative growth rate of each trout as ln*S*_*2*_ – lnS_*1*_, where *S*_*2*_ is the mass in grams or fork length in mm after 3, 6, and 9 weeks, and S_*1*_ is the mass or fork length at the start of the experiment. We analysed differences in growth rate between infested and non-infested trout using a full-factorial general linear model with growth rate as the dependent variable, infestation / no infestation at the start of the experiment as a fixed factor, experimental aquarium as a random factor, and initial trout size as a covariate. We ensured that growth data, both for mass and length, met assumptions of normality and homoscedasticity.

We also tested how glochidia encystment (the total number of glochidia and the number of glochidia standardized by trout mass) on infested trout changed over time using repeated-measures ANOVAs, with glochidia abundance at 0, 3, 6, and 9 weeks as a within-subject factor. We used a Greenhouse-Geisser correction, as the data did not meet the assumption of sphericity. For infested trout, we also tested how the number of sloughed glochidia (i.e., the difference in glochidia load between the end and the beginning of the experiment) varied with relative growth rate, using Pearson correlation.

Among the infested trout, we noted two distinctive groups, where 10 trout individuals had a decreasing number of glochidia over time, whereas 10 trout individuals did not (the four remaining infested trout died before the experiment was finalized). Hence, we also tested for differences in the relative growth rate (based on length and mass, respectively) between these groups using t-tests, as the data did not deviate from normality and homoscedasticity. All statistical tests were done in IBM SPSS 26 (Armonk, NY, USA). We counted the number of glochidia on the trout as exactly as possible. At glochidia loads > 100, this was not feasible, however. Therefore, glochidia loads of ≥ 100 were set to 100 in all analyses.

## Results

### Prey handling time

**Fig. 1:**
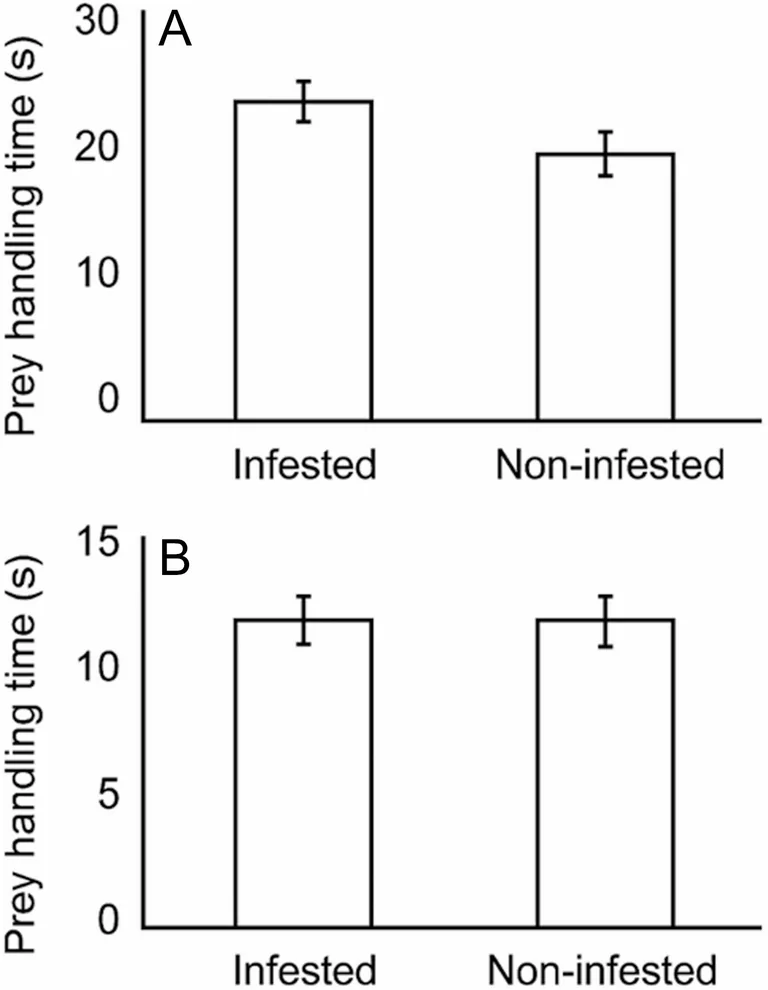
Prey-handling time of infested and non-infested juvenile brown trout (*Salmo trutta*) preying on maggots (*Calliphoria vomitaria*) (**A**) and *Gammarus pulex* (**B**). Error bars denote ± 1 standard error

Infested trout generally took a longer time to consume the maggots compared to non-infested trout (Wald *χ*^*2*^ *= 4.37*,* df = 1*,* p* = 0.037) (Fig. 1A), and trout mass did not seem to influence the handling time of maggots (Wald *χ*^*2*^ *= 0.14*,* df = 1*,* p* = 0.71). In contrast, the time to consume *G. pulex* did not differ between infested trout and non-infested trout (Fig. 1B), (Wald *χ*^*2*^ *= 0.01*,* df = 1*,* p* = 0.93), and trout mass did not influence the handling time of *G. pulex* (Wald *χ*^*2*^ *= 0.75*,* df = 1*,* p* = 0.39).

### Growth

**Fig. 2:**
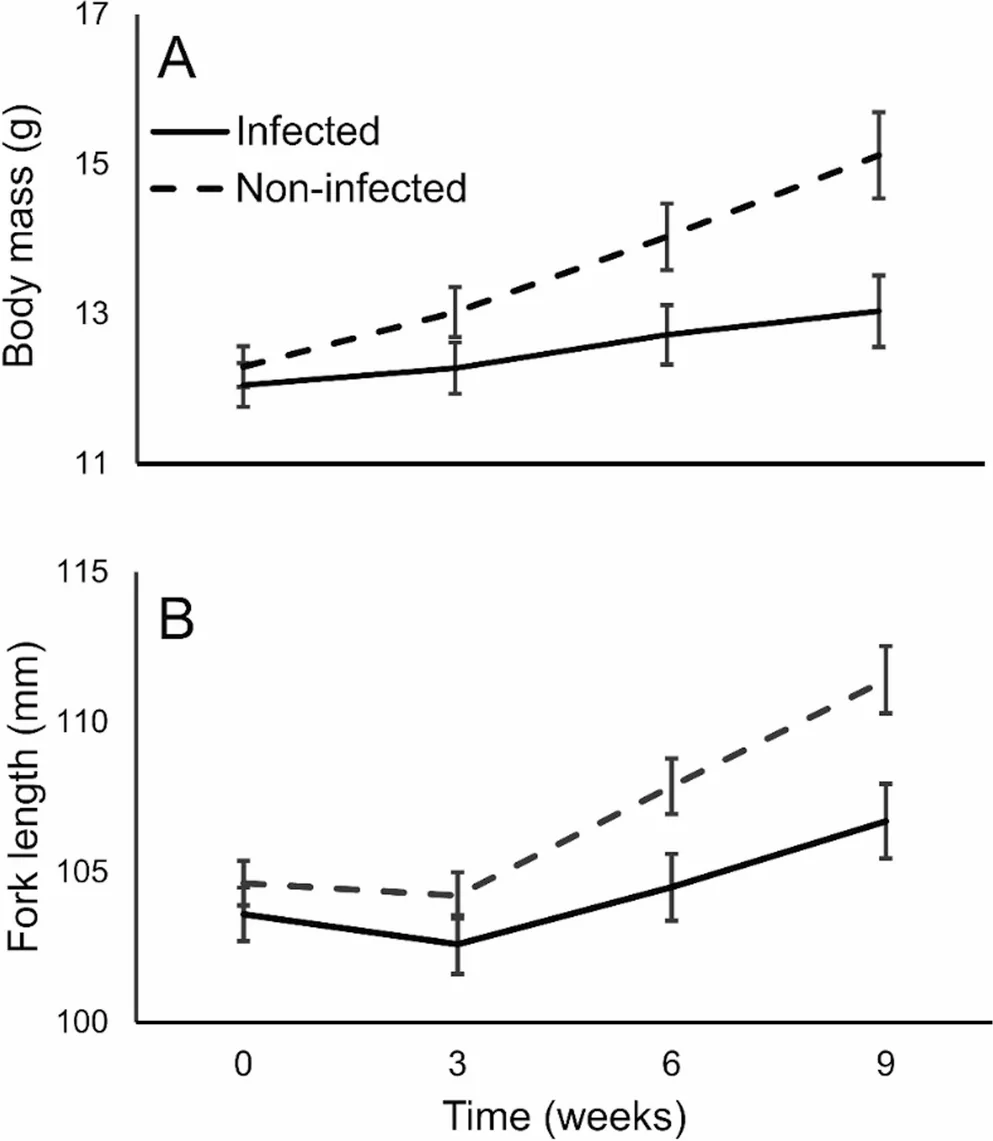
Body mass in grams (**A**) and fork length in mm (**B**) of juvenile brown trout (*Salmo trutta*), infested and non-infested with parasitic freshwater pearl mussel (*Margaritifera margaritifera*) glochidia, during nine weeks in experimental aquaria. Error bars denote ± 0.5 standard error (changed from ± 1 for visibility)

**Fig. 3:**
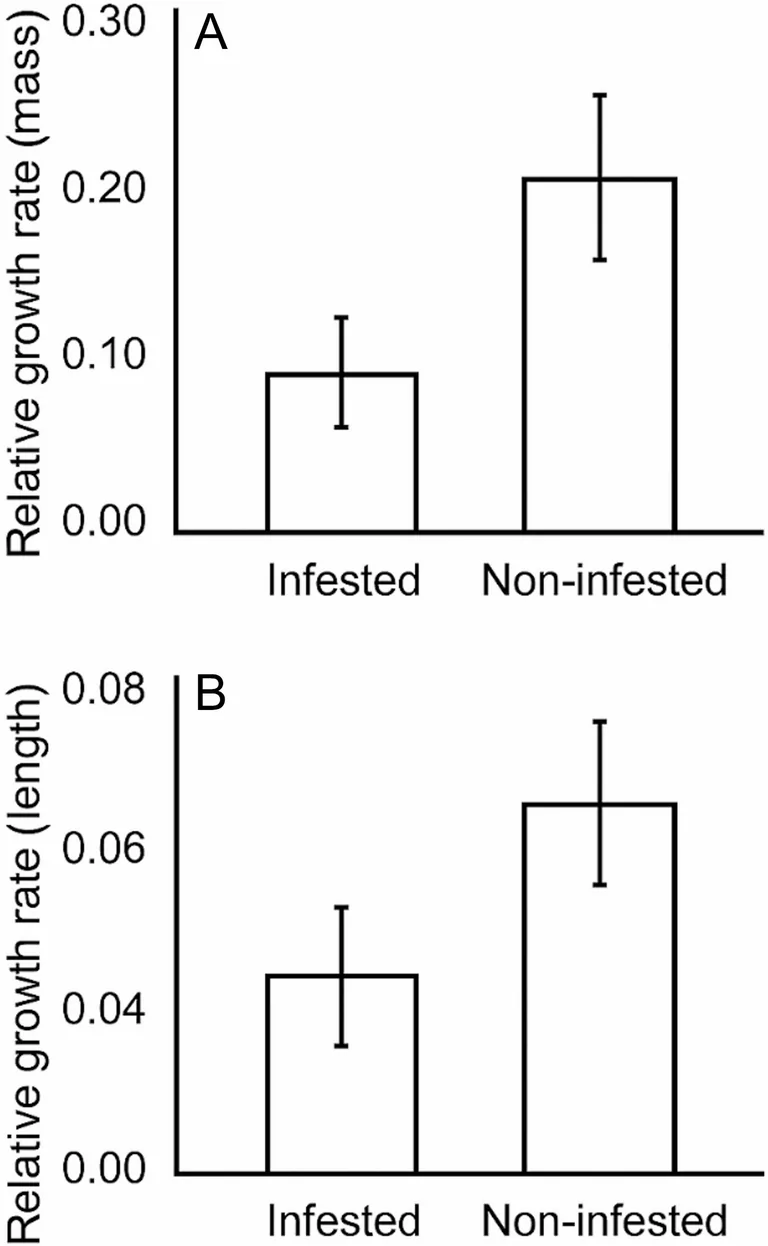
Relative growth rate based on mass (g) (**A**), and fork length (mm) (**B**) of juvenile brown trout (*Salmo trutta*) infested and non-infested with parasitic freshwater pearl mussel (*Margaritifera margaritifera*) glochidia. Error bars denote standard error

Both infested and non-infested brown trout increased in size during the experiment, albeit non-infested trout tended to grow faster (Fig. 2A-B). Infested trout had a lower growth rate based on weight than non-infested trout (*F*_*1, 16.6*_ = 6.39, *η*_*p*_^*2*^ = 0.28, *p* = 0.022) (Fig. 3A). A similar trend was observed for relative growth rate based on length (*F*_1, 29.7_ = 3.57, *η*_*p*_^*2*^ = 0.107, *p* = 0.069) where infested trout had a lower relative growth rate compared with non-infested trout (Fig. 3B). Neither experimental aquaria (*p* > 0.8 in both cases) nor initial trout size (*p* > 0.9 in both cases) had a significant effect on trout growth.

**Fig. 4:**
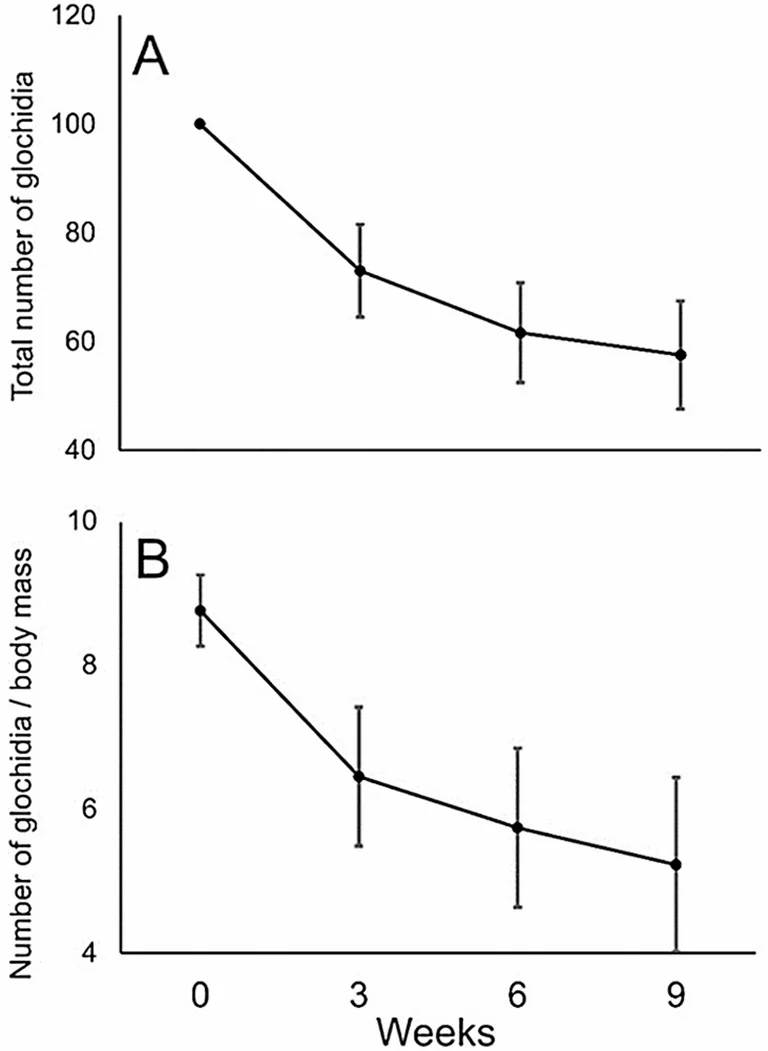
Mean number of glochidia (**A**), and mean number of glochidia divided by trout body mass (glochidia/g) (**B**) on the gills of infested trout over time during a nine-week growth experiment. Error bars denote ± 1 standard error

**Fig. 5:**
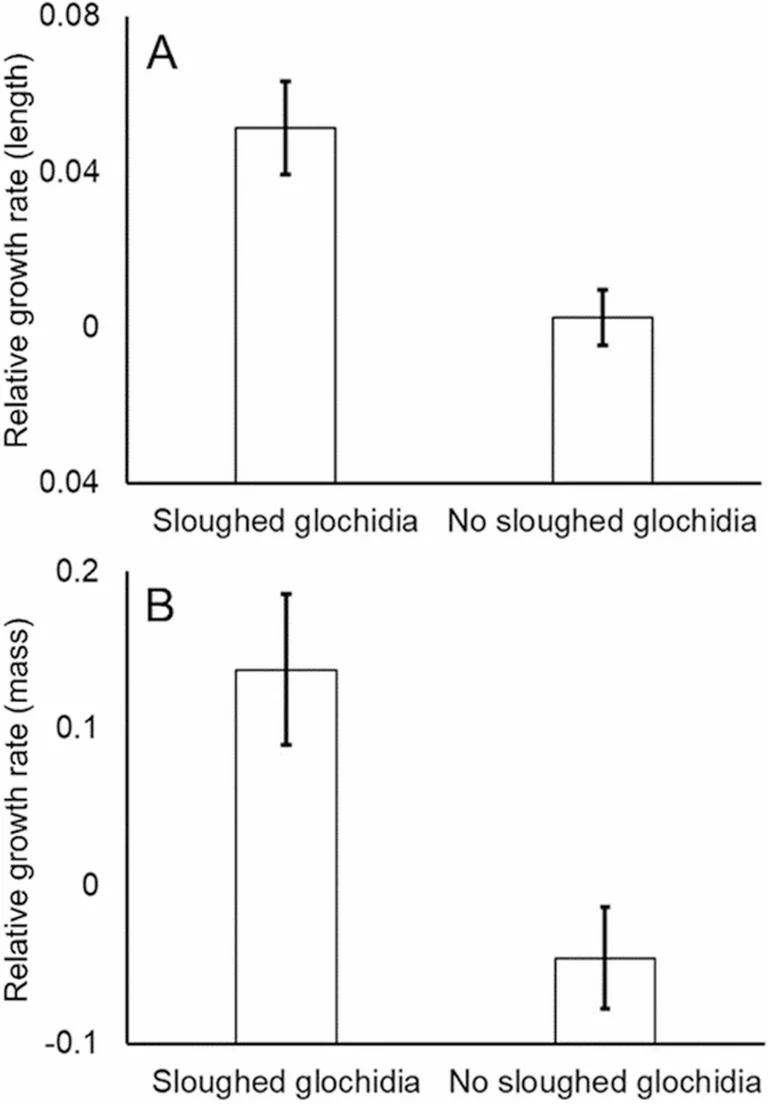
Relative growth rate based on length (mm) (**A**), and body mass (g) (**B**) of juvenile brown trout (*Salmo trutta*) during a nine-week-long growth experiment. The figure compares brown trout that sloughed freshwater pearl mussel (*Margaritifera margaritifera*) glochidia, and thus reduced their glochidia load over time, to infested trout that did not slough any glochidia. Error bars denote ± 1 standard error

The infection intensity decreased over time for the total number of glochidia (*F*_3, 37_ = 9.71, *η*_*p*_^*2*^ = 0.39, *p* < 0.001) and when glochidia abundance was standardized per trout mass (*F*_3, 35_ = 9.82, *η*_*p*_^*2*^ = 0.34, *p* = 0.001) (Fig. 4). The relative growth rate based on length was positively correlated with the number of sloughed glochidia over the experiment (*r* = 0.45, *p* = 0.046), while no such correlative pattern was found for relative growth rate based on mass (*r* = 0.37, *p* = 0.11). Trout that lost glochidia during the experiment had a higher growth rate based on length than trout that did not lose any glochidia (T_18_ = -2.35, *p* = 0.031).) (Fig. 5A). We also found the same significant pattern for relative growth rate based on body mass (*T*_18_ = -2.13, *p* = 0.048), where trout that lost glochidia over time had a higher growth rate (mean ± se, 0.137 ± 0.048) than trout that did not lose any glochidia (mean ± se, -0.046 ± 0.032) (Fig. 5B).

## Discussion

In agreement with our hypotheses, the prey handling time of large prey for trout held individually was longer, while growth in groups was lower for infested than for non-infested trout. Our findings support results from previous studies on individual foraging efficiency, where infested trout caught 20% fewer prey items than non-infested trout (Österling et al. 2014), while high loads of glochidia reduced the competitive ability in pairwise trials between infested and non-infested brown trout (Filipsson et al. 2016). Previous experiments have also shown that artificial infestation can reduce trout growth by up to 10%, possibly caused by increases in respiratory burden and/or immune responses, both energetically costly physiological processes (Chowdhury et al. 2019). Our results suggest that reduced prey handling caused by glochidia infestation can lead to reduced energy intake on the individual level, which can ultimately be a reason behind reduced growth of infested trout competing for the same food resource when living in groups with non-infested trout.

Reductions in energy intake and growth of infested trout probably influence their fitness and life history tactics in the wild. For example, growth is a trigger for outward migration in anadromous fish, and low growth of infested trout might delay their downstream migration or increase the proportion of resident individuals (Bohlin et al. 1996). Such behavioral changes may be transitory in trout that lose glochidia over time, since we found that their growth was higher than that of trout that did not lose glochidia. One main reason for glochidia loss is the strength of the trout’s immune reaction, which is also costly for the fish. However, it seems that trout gain more by having a strong and costly immune defence against glochidia infestations (Klemme and Karvonen 2016; Chowdhury et al. 2019), since the infested trout that lost glochidia in our study seemed to have compensatory growth, which may reduce the negative effects of glochidia infestation on trout fitness in the wild.

Our results showed that infested trout feeding on large maggots increased their prey handling time compared to non-infested trout. In contrast, the prey handling time for the smaller *G. pulex* was almost identical between infested and non-infested trout. We believe that the glochidia infestation was negatively affecting food intake, since infested and non-infested trout did not differ in size and were kept separately in different aquaria. The mechanism behind the phenomenon is unknown, but a change in gill morphology, where gills and gill rakers become thicker and longer when infested with glochidia (Thomas et al. 2013), may hinder trout from feeding normally (inertial sucking). Although the size difference between maggots and *G. pulex* was not observed to differ, the prey types differed substantially in morphology. Maggots are soft-bodied, flexible, and continue moving after capture, whereas *G. pulex* is more compact and rigid due to its exoskeleton (Madrell, 2018; Trevisan et al. 2014). These differences may influence the time required for prey manipulation and ingestion and could therefore contribute to differences in prey-handling times. Lastly, although ethical and logistical challenges in sampling wild fish limited our sample sizes, the observed differences show strong trends suggesting a biologically relevant impact of parasite infestation.

Significant growth differences between infested and non-infested trout could only be seen for growth based on mass and not for growth based on length. This agrees with previous findings where length is generally more fixed and takes a longer time to change, in contrast to changes in weight (Fuentes et al. 2013). For infested fish that lose their glochidia larvae over time, the relative growth rate was higher both based on weight and length. Most likely, compensatory growth will enhance an individual’s status in a population and their competitive ability towards conspecifics in the wild. However, compensatory growth has also been shown to have costs, such as an increased risk of being eaten by predators (Gotthard 2000) or a reduced lifespan (Ozanne and Hales 2004).

In conclusion, we suggest that natural glochidia infestation may impair the competitive ability and increase the prey handling time of juvenile brown trout, possibly reducing trout growth and fitness. While different food types were used across experiments and food intake was not directly measured during the growth trial, the prolonged handling times observed with natural prey suggest a plausible mechanism for the reduced growth. Our results agree with studies using artificially infested fish, which show negative effects on trout (Taeubert and Geist 2013; Thomas et al. 2013; Chowdhury et al. 2019). Thus, our results suggest that glochidia infection may decrease the fitness of brown trout also under natural conditions in the wild (Blanchet et al. 2009). Lastly, our results indicate that some trout individuals seem to fend off glochidia infestations over time, while others cannot, and that this influences growth. To what extent these differences are related to individual and innate differences in phenotypic traits needs to be tested, and the ecological effects validated in the wild. 

## Data Availability

No datasets were generated or analysed during the current study.

## Declarations

### Open access

I confirm that I understand Parasitology Research is an open access journal that levies an article processing charge per articles accepted for publication. By submitting my article I agree to pay this charge in full if my article is accepted for publication.

### Dual publication

The results/data/figures in this manuscript have not been published elsewhere, nor are they under consideration by another publisher.

### Third-party material

All of the material is owned by the authors and/or no permissions are required.
